# Genome-wide expression patterns of invasion front, inner tumor mass and surrounding normal epithelium of colorectal tumors

**DOI:** 10.1186/1476-4598-6-79

**Published:** 2007-12-14

**Authors:** Eike Staub, Joern Groene, Maya Heinze, Detlev Mennerich, Stefan Roepcke, Irina Klaman, Bernd Hinzmann, Esmeralda Castanos-Velez, Christian Pilarsky, Benno Mann, Thomas Brümmendorf, Birgit Weber, Heinz-Johannes Buhr, André Rosenthal

**Affiliations:** 1 Max Planck Institute for Molecular Genetics, Department of Computational Molecular Biology, Berlin, Germany; 2 Merck Serono, Bio- & Chemoinformatics, Darmstadt, Germany; 3 metaGen Pharmaceuticals i.L., Berlin, Germany; 4 Charité – Campus Benjamin Franklin, Department of General, Vascular and Thoracic Surgery, Berlin, Germany; 5 Boehringer Ingelheim Pharma GmbH & Co KG, Biberach, Germany; 6 Signature Diagnostics, Potsdam, Germany; 7 Epigenomics, Berlin, Germany; 8 University Hospital Carl Gustav Carus Dresden, Department of Visceral, Thoracic, and Vascular Surgery, Germany; 9 Augusta-Kranken-Anstalt GmbH, Department of Surgery, Bochum, Germany; 10 Novartis Institutes for BioMedical Research, Novartis Pharma AG, Basel, Switzerland; 11 Nycomed, Konstanz, Germany; 12 ratiopharm, Ulm, Germany

## Abstract

Colorectal tumors have characteristic genome-wide expression patterns that allow their distinction from normal colon epithelia and facilitate clinical prognosis. The expression heterogeneity within a primary colorectal tumor has not been studied on a genome scale yet. Here we investigated three compartments of colorectal tumors, the invasion front, the inner tumor mass, and surrounding normal epithelial tissue by microdissection and microarray-based expression profiling. In both tumor compartments many genes were differentially expressed when compared to normal epithelium. The sets of significantly deregulated genes in both compartments overlapped to a large extent and revealed various interesting known and novel pathways that could have contributed to tumorigenesis. Cells from the invasion front and inner tumor mass, however, did not show significant differences in their expression profile, neither on the single gene level nor on the pathway level. Instead, gene expression differences between individuals are more pronounced as all patient-matched tumor samples clustered in close proximity to each other. With respect to invasion front and inner tumor mass we conclude that the specific tumor cell micro-environment does not have a strong influence on expression patterns: largely similar genome-wide expression programs operate in the invasion front and interior compartment of a colorectal tumor.

## Background

Recent reports have highlighted differences between the invasion front and interior compartments of colorectal tumors. Tumor cell budding at the invasion front is increasingly recognized as an adverse prognostic factor for colorectal tumors [1,2]. Hypoxia in the inner tumor mass is thought to induce angiogenesis through changes in hypoxia inducible factor-regulated gene transcription [3] and to induce epithelial-mesenchymal transition [4]. Downstream targets of the Wnt/beta-catenin pathway and beta-catenin itself exhibit stronger protein expression at the invasion front of colon tumors [5-8]. However, recent success in the prediction of metastatic properties of tumors from genome-wide expression profiles of whole tumors [9,10] suggest that the capability to metastasize is imprinted in the expression programs of the majority of primary tumor cells, not only those at the invasion front. It is not clear whether the reported enhanced protein expression of some Wnt/beta-catenin pathway targets is also reflected on the level of transcription. In general, little is known about how similar the genomes of cells in different tumor compartments are and even less is known about the differences in their gene expression programs.

This motivated us to study the expression of the invasion front, inner cells and surrounding normal epithelia of primary colorectal tumors by laser-capture microdissection and genome-wide microarray expression analysis. To our knowledge this is the first study of expression heterogeneity of a primary tumor on a genome scale. Cells from distinct tumor compartments were separated by laser-capture microdissection (documented electronic images are available upon request). RNA amplification was necessary to obtain sufficient starting material for hybridization of Affymetrix U133A DNA chips. For methodological details we refer to our previous work [11-13]. We investigated the expression data by unsupervised multivariate data mining techniques, statistical group testing and further explored the results by the use of different pathway/gene group analysis algorithms and databases.

## Results and discussion

**Figure 1:**
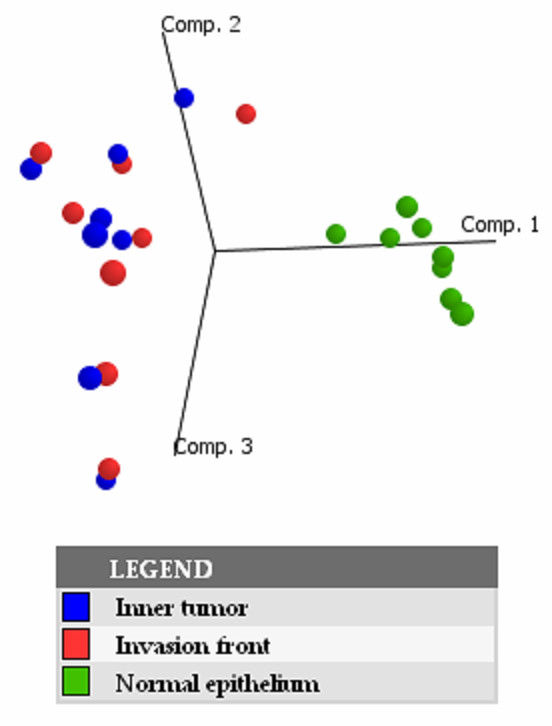
Principal component analysis of tumor samples from the invasion front (IT), the inner tumor mass (RT) and from neighbored normal epithelium (N) based on the expression patterns of 7433 genes. Note that normal epithelia cluster closely together to the exclusion of all tumor samples.

**Figure 2:**
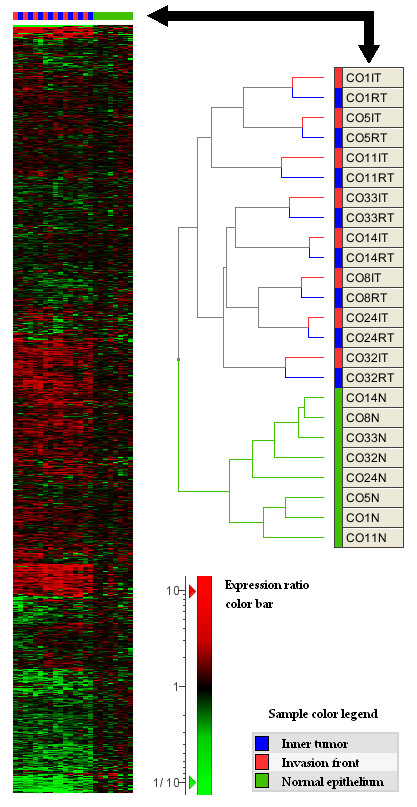
Two-way hierarchical clustering of tumor and normal epithelia samples based on expression profiles of 7433 genes. (A) The heat map shows expression changes relative to the average signal in normal tissues. Red means up-regulation, green means down-regulation (see expression ratio color bar at the bottom). (B) The dendrogram shows the hierarchical order of similarities between patient samples. Note that all normal samples are separated from tumors and both samples of a patient that stem from different tumor compartments clustered as neighbors.

Much information about dominant trends in high-dimensional gene expression data can be discovered by the application of dimension reduction techniques. We applied principal component analysis on the covariance matrix to analyze major trends in our data (see Figure 1). The first three principal components (Eigengenes) capture >43% of the variance of all genes and therefore are suspected to reflect major expression trends in the data set. In the 3D plot of the first three principal components we found that samples of normal epithelia clustered together and were well separated from all tumor samples. Moreover the tumor samples tend to localize in pairs, with one sample from the invasion front next to a matching sample from the inner tumor mass. This result was confirmed by hierarchical clustering (see Figure 2) of the tumor samples according to the similarity of their global gene expression patterns. Matching tumor samples from the invasion front and inner tumor compartments clustered as direct neighbors whereas all samples from normal epithelia were separated from their matching tumors. We conclude that genome-scale expression signals are different between normal colorectal epithelia and carcinoma cells. But expression differences between invasion front and inner tumor mass are not strong enough to overcome the expression variability associated with individual tumors. In other words, with respect to the two analyzed compartments the within-tumor gene expression variability is lower than the between-tumors expression variability.

The analyses so far neither do reveal how strong gene expression differences between tumor and normal cells are, nor do they allow to conclude that there are any significant expression changes between tumor compartments at all. To investigate the strengths of expression differences among sample groups we performed statistical tests. In comparisons of the normal epithelium with interior tumor cells we found 1528 of 7433 genes under investigation (see Additional files 1 &2) to be differentially expressed (by both, unpaired Welch t-tests and paired t-tests at an FDR threshold of Q<0.1 according to the Benjaminin-Hochberg method). When comparing the normal epithelium with matching tumor invasion fronts we found 1128 genes to be differentially expressed according to both tests. The lists of differentially expressed genes are strongly overlapping: 923 genes (>80% of the smaller list) are differentially regulated in both tumor compartments (a full list of all genes, their expression data and annotations, and their statistical test results is available in Additional files 1 &2). This analysis proves that there are enormous differences in mRNA expression between colorectal carcinoma cells and their matching epithelia with more than 10% of all expressed genes being affected. The majority of these genes are deregulated in both compartments of colorectal tumors, the invasion front and the inner cell mass.

Does the functional annotation of the 923 commonly deregulated genes tell us something about cellular processes involved in colorectal tumor development? We clustered all differential genes (k-means; Euclidean distance; k = 2) into two clusters containing 508 up-regulated and 415 down-regulated genes. Both sets of genes were tested separately for significant overlap with gene sets from public databases using Fisher's Exact test (see Additional file 3). These databases capture information on signaling pathways and their downstream targets, clinical prognosis gene sets, genomic cancer gene neighborhoods and cellular structures and mechanisms. We found that a very high number of gene sets is over-represented among the 501 up-regulated genes (148 categories at p < 0.0004 and Q<0.01). Among these are many categories linked to proliferation or development of neoplastic processes, a functional link that has been described before numerous times [14]. Surprisingly, we found several gene groups linked to proteasomal degradation to be over-represented among the up-regulated genes. This highlights the important role of the proteasome in cancer development that was recently proven by the development of the proteasome inhibitor Bortezomib [15]. Our results suggest that proteasome inhibition could also be beneficial for the treatment of colorectal cancer especially in patients with up-regulated proteasomal genes. Furthermore, we found that gene sets previously described to be down-regulated in response to Rapamycin or HDAC inhibitors are over-represented among our 501 tumor up-regulated genes [16,17]. This suggests that such drugs have the potential to partially revert the gene expression patterns that emerge during colorectal tumorigenesis. Also clinical prognosis-relevant gene sets were overrepresented among up-regulated genes, e.g. the breast cancer prognosis signature of van't Veer and coworkers that shares many genes with typical proliferation signatures [14,18]. The top scoring gene group, however, was the coexpression neighborhood of the cancer gene RAN in an expression atlas of human tissues [19] which suggests a prominent role for this gene group in our cancer patients. The functional annotation of down-regulated genes in colorectal tumors resulted in a smaller number of significant functional categories: only 20 categories were over-represented at a relaxed threshold of p < 0.0006 (Q<0.05). Interestingly, in our set of colorectal tumors a similar set of genes is downregulated as that found by Sansom and coworkers after induction of mutant APC in a mouse model [20]. APC is a well known component of the Wnt/beta-catenin pathway. The Wnt/beta-catenin pathway is often hyper-activated by mutation in colorectal cancers. This finding suggests that the Wnt/beta-catenin pathway may also be deactivated in our set of tumors. It raises the question whether loss rather than gain of expression upon Wnt/beta-catenin pathway activation is crucial for colorectal cancer development. In summary, the investigation of functional annotations of deregulated genes recovered some known functional aspects of colorectal tumors and highlights several novel routes to explore colorectal cancer biology and treatment.

**Figure 3:**
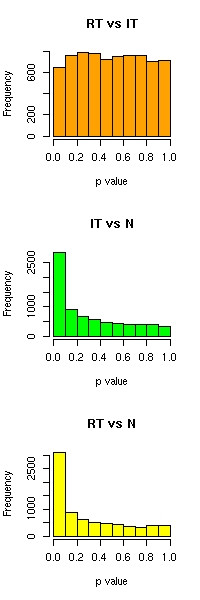
Histograms of p-value distributions for pair-wise comparisons between three tissue groups. Note that the p-value distributions for gene-wise comparisons between tumor compartments (RT, IT) and normal epithelia (N) have a peak at p-values approaching 0 that is typical for situations in which multiple genes have highly significant p-values (like tumor-normal comparisons). In contrast, the p-value distribution of the invasion front (IT) vs. inner tumor (RT) comparison lacks this peak and p-values are uniformly distributed, just as if one would have labeled the experiments randomly. This means that there are no gross differences in gene expression between the two tumor compartments.

The instructive comparison of gene expression patterns of tumor and normal samples prompted us to investigate gene expression differences between tumor cells from the invasion front and inner tumor mass. However, neither paired t-tests nor unpaired Welch t-tests resulted in single genes with significant differences between samples of the two compartments. Single top genes reached p-values of 0. 003 in the Welch t-test and 0.0005 in the paired t-test: both results cannot be regarded as significant when one considers the large number of tests (FDR>0.98 when using these p-values as thresholds). In histograms showing the distribution of p values (see Figure 3) we found that the invasion front-inner tumor comparison yielded much less low range p-values compared to the tumor-normal comparisons. Recently, novel statistical methods such as Gene Set Enrichment Analysis (GSEA) were developed to detect subtle expression changes in functionally-related gene groups in the absence of positive test results for individual genes: these methods led to the discovery of molecular signatures for diabetic and aging muscle [21-23]. We applied the Tian *et al*. variant of GSEA using gene groups supplied by the GO, KEGG, GenMAPP and MSIGDB databases. However, even this sensitive algorithm did not deliver a single gene group that can be regarded as differentially expressed. We therefore conclude that on the genome scale there are no significant differences in mRNA expression patterns between colorectal tumor invasion front and inner tumor mass. It appears that cells from these compartments of a single primary colorectal tumor are equipped with similar transcriptomes. This suggests that they may also be equipped with largely similar genomes. In the light of numerous reports on differentially expressed proteins at the colorectal tumor invasion front, in particular Wnt/beta-catenin pathway components, we speculate that the abundance of such proteins is primarily regulated on the posttranscriptional level, possibly by stabilization of pathway components and pathway downstream targets that are induced by interaction with tumor cells and stroma at the invasion front.

## Conclusion

We investigated the invasion front, the inner tumor mass, and surrounding normal epithelial tissue of colorectal tumors by laser-capture microdissection and microarray-based expression profiling. In comparisons of both tumor compartments with normal epithelium many genes were differentially expressed. The lists of deregulated genes in both compartments overlapped to a large extent. In contrast, cells from the invasion front and inner tumor mass did not show significant differences in their expression profile, neither on the single gene level nor on the pathway level. We conclude that largely similar genome-wide expression programs operate in the invasion front and interior compartment of a colorectal tumor.

## Methods

### Sample acqusition

Unselected consecutive CRC patients undergoing elective standard oncological resection at the Department of General, Vascular and Thoracic Surgery, Campus Benjamin Franklin, Charite, were prospectively recruited. The study was approved by the local ethical committee and informed consent was obtained from all patients.

### Laser-capture microdissection

Preprocessing of frozen tissue blocks by laser-capture microdissection was essentially performed as described in our previous publications [11-13]. Briefly described, all cancer specimens were snap frozen within 20 min following excision and snap frozen. During laser-capture microdissection frozen tissue specimens were serially cut into 6–8-mm-thick sections which were mounted on a sterile 2.5 mm membrane. Slides were fixed in 70% ethanol. The sections were briefly stained with haematoxylin and eosin, dehydrated in ethanol and dried for 10–15 min using an excicator. The membrane was turned around and fixed with adhesive tape on the other sterile slide. First slides served as a template on which the areas of tumour or normal epithelium were marked. On the consecutive section, these areas were microdissected using a laser microdissection system (SL, Jena, Germany and P.A.L.M. Microlaser Technologies AG Bernried, Germany) and capture transfer films (Arcturus GmbH, Moerfelden-Walldorf, Germany). For molecular analysis, up to 100,000 cells or approximately 30–60 mm^2 ^of tissue section areas were pooled and collected in ice-cooled tubes containing 100 ml of 98% guanidine thiocyanate (GTC) buffer and 2% beta-mercaptoethanol. For molecular analysis, up to 100,000 cells were pooled. From each tumor we isolated cell populations of the invasion front, the inner cell mass and normal epithelium from the periphery.

### Messenger RNA preparation and DNA chip hybridization

Poly AC RNA from the microdissected specimens was prepared using the PolyA-tract 1000 kit (Promega, Heidelberg, Germany) according to the manufacturer's recommendations. For each sample, the cDNA synthesis and repetitive in vitro transcription were performed three times. The total amount of prepared mRNA from each sample was used. First strand cDNA synthesis was initiated using the Affymetrix T7-oligo-dT promoter-primer combination at 0.1 mM. The second strand cDNA synthesis was generated by internal priming. In vitro transcription was performed using Ambion's Megascript kit (Ambion, Huntington, UK) as recommended by the manufacturer. From the generated cRNA, a new first strand synthesis was initiated using 0.025 mM of a random hexamer as primer. After completion, the second strand synthesis was performed using the Affymetrix T7-oligo-dT promoter-primer combination. A second in vitro transcription was performed and then the procedure was repeated one additional time. During the last in vitro transcription, biotin-labelled ribonucleotides were incorporated into the aRNA, as recommended by the Affymetrix protocol. Hybridisation and detection of the labelled cRNA on the Affymetrix Chip were performed according to Affymetrix standard protocol.

### Microarray data preprocessing

Raw expression data was condensed to probe set-wise intensity values using the GC-RMA algorithm. Present/absent calls for each data point were calculated with the Affymetrix standard algorithm. Only genes with significant expression signals (Affymetrix "present calls" at alpha = 0.04) in a minimum of 30% of experiments were considered for subsequent analyses. For experiment normalization data were rescaled to have equal median expression intensities for all experiments, then intensities for each gene were divided by the average over all experiments with normal tissues and the logarithm of this ratio was calculated. Probe set annotation for U133A chips were retrieved from the Affymetrix web site (version 22). Gene-wise expression data were obtained by averaging intensities of all probe sets associated with a gene symbol, finally resulting in log ratio expression data for 7433 genes.

### Expression data analysis

The preprocessed data were subjected to a principal component analysis (PCA) of the covariance matrix of the data to investigate and visualize global trends in the data. Hierarchical clustering using the complete linkage algorithm and Euclidean distances was used as a complementary method to analyze the grouping of samples. These algorithms were used as implemented in the Expressionist Analyst software (Version 4.1, Genedata, Basel). Pairwise Welch t-tests were performed for each gene to compare expression levels in the the sample groups invasion front (IT), inner tumor mass (RT) and normal epithelial tissue (N). Histograms of all resulting p values for each comparison were plotted using the statistical software R. The p value distributions helped to assess whether there are differences in expression on the genome level between the three sample groups. If there are no significant differences in expression (comparable to a situation when the data are randomized) the p value distribution resembles a uniform distribution.

## Competing interests

The author(s) declare that they have no competing interests.

## Authors' contributions

ES performed data analysis and drafted the manuscript, JG and MH performed RNA sample preparation, microdissection and hybridization of chips, DM hybridized DNA chips and contributed to data analysis, SR was involved in data preprocessing and analysis, IK performed laser capture microdissection, ECV did laser capture microdissection, TB supervised chip hybridization and data preprocessing, BM and HJB were responsible for clinical part of the study including sample acquisition and patients' informed consent, CP and BW supervised chip hybridization, quality control and data preprocessing, AR conceived the study.

## Supplementary Material

Additional File 1 Microarray expression data of 7433 genes. The pre-processed expression data for all 7433 genes that were considered for subsequent analysis. The data are logarithms (base e) of expression ratios between each measurement and the mean value over all experiments with normal epithelia. Therefore, these log ratios indicate relative changes compared to normal epithelia. Positive values indicate up-regulation compared to normal epithelia. For further preprocessing steps see legend of Figure 1 in the manuscript. Click here for file

Additional File 2 Results from statistical tests for differential expression. These tables comprise results from statistical testing for differential gene expression and the annotations for all 7433 genes. The gene-wise p-values and Q-values of unpaired Welch test or the paired t-test (Benjamini-Hochberg method) for each sample group comparison are listed. The gene group memberships of each gene are separated by a colon in the respective column. KEGG, GenMAPP and GO categories for all genes were taken from the Affymetrix U133A DNA chip annotation . All other gene groups were taken from the MSIGDB database . Click here for file

Additional File 3 Significantly deregulated gene groups. Here we show the results of Fisher's Exact test for the over-representation of functional gene groups among up- or down-regulated genes in colorectal tumors of our study. To correct for multiple testing Q-values were calculated using the Benjamini-Hochberg method. Click here for file
